# QTc prolongation across CDK4/6 inhibitors: a systematic review and meta-analysis of randomized controlled trials

**DOI:** 10.1093/jncics/pkae078

**Published:** 2024-09-10

**Authors:** Bruno Murad, Pedro C A Reis, Alice Deberaldini Marinho, Ana Carolina Marin Comini, Débora Pinheiro Xavier, Beatriz Mella Soares Pessoa, Farah Raheem, Brenda Ernst, Lida A Mina, Felipe Batalini

**Affiliations:** Faculdade de Medicina de Barbacena (FUNJOB), Minas Gerais, Brazil; Universidade Federal do Rio de Janeiro, Rio de Janeiro, Brazil; Universidade Federal do Estado do Rio de Janeiro (UNIRIO), Rio de Janeiro, Brazil; A. C. Camargo Cancer Center, São Paulo, Brazil; Universidade Federal do Pará (UFPA), Pará, Brazil; University of Connecticut, Farmington, CT, USA; Mayo Clinic, Phoenix, AZ, USA; Mayo Clinic, Phoenix, AZ, USA; Mayo Clinic, Phoenix, AZ, USA; Mayo Clinic, Phoenix, AZ, USA

## Abstract

**Background:**

Cyclin-dependent kinases (CDK) 4/6 inhibitors have significantly improved outcomes for patients with ER+/HER2− breast cancer. Nevertheless, they differ from each other in terms of chemical, biological, and pharmacological features, as well as toxicity profiles. We aim to determine whether QTc prolongation is caused by CDK4/6i in general or if it is associated with ribociclib only.

**Methods:**

We systematically searched PubMed, Embase, and Cochrane Library for randomized controlled trials (RCTs) comparing the prevalence of QTc prolongation as an adverse event in HR+ breast cancer patients treated with CDK4/6i vs those without CDK4/6i. We pooled relative risk (RR) and mean difference (MD) with 95% confidence interval (CI) for the binary endpoint of QT prolongation.

**Results:**

We included 14 RCTs comprising 16 196 patients, of whom 8576 underwent therapy with CDK4/6i. An increased risk of QTc prolongation was associated with the use of CDK4/6i (RR = 2.35, 95% CI = 1.67 to 3.29, *P* < .001; I^2^ = 44%). Subgroup analyses revealed a significant increase in the QTc interval for the ribociclib and palbociclib cohorts. The ribociclib subgroup showed a relative risk of 3.12 (95% CI = 2.09 to 4.65, *P* < .001; I^2^ = 12%), whereas the palbociclib subgroup had a relative risk of 1.51 (95% CI = 1.05 to 2.15, *P* = .025; I^2^ = 0%).

**Conclusion:**

Palbociclib was associated with QTc prolongation; however, the relative risk for any grade QTc was quantitively twice with ribociclib. Furthermore, grade 3 QTc prolongations were observed exclusively with ribociclib. These results are important for guiding clinical decision-making and provide reassurance regarding the overall safety profile of this drug class.

The activation of cyclin-dependent kinase (CDK) 4/6 through cyclin D leads to the phosphorylation and subsequent deactivation of the tumor suppressor protein Retinoblastoma protein (Rb). This phosphorylation event enables cells to progress from the G1 phase to the S phase, promoting DNA replication and subsequent cell division. In cancer, mutations or overexpression of CDK4/6 and cyclin D can instigate excessive cell proliferation (1,2). Targeting CDK4/6 can reestablish Rb’s tumor-suppressive role, effectively halting uncontrolled cell growth. This makes targeting CDK4/6 an appealing therapeutic strategy (3).

The CDK4/6 inhibitors selectively target CDK4/6, preventing progression through this checkpoint. This action results in cell cycle arrest, effectively reducing cancer cell proliferation (4). Notably, CDK4/6 inhibitors have demonstrated remarkable efficacy in specific cancer types, particularly in estrogen receptor-positive (ER+) breast cancer (5). Three CDK4/6 inhibitors have received Food and Drug Administration (FDA) approval and have been integral in advancing cancer treatment (6,7). Palbociclib was the first CDK4/6 inhibitor to receive FDA approval in 2015. Palbociclib is used in combination with endocrine therapy and has significantly improved progression-free survival in ER+, metastatic breast cancer patients (8). Ribociclib (Kisqali) was FDA approved in 2017 in combination with endocrine therapy for ER+, metastatic breast cancer. Clinical trials have demonstrated its efficacy in delaying disease progression and improving overall survival (9,10). Abemaciclib (Verzenio) was FDA approved in 2017. Abemaciclib differs from the other CDK4/6 inhibitors because it can be used as a single agent or in combination with hormonal therapy. Dalpiciclib, as of January 2024, has not received FDA approval. However, it has demonstrated promising outcomes in combination with anti-estrogenic therapy for treating hormone-receptor positive advanced breast cancer (11). Trilaciclib is approved for reducing chemotherapy-induced myelosuppression in extensive-stage small-cell lung cancer (12). Promising results in trials for metastatic triple-negative breast cancer indicate increased overall survival, but trilaciclib is not currently used in hormone receptor-positive breast cancer (13).

Recent reports and studies have raised concerns regarding CDK4/6i and cardiovascular adverse events (14). Specifically, QTc prolongation has been correlated to ribociclib, such as the FDA recommending against the combination of ribociclib and TAM advised by the findings of the MONALESSA-7 study (15). In the pooled analysis of the MONALEESA trials, 5.6% of patients in the ribociclib group experienced QT prolongation vs 1.5% of patients in the placebo group (16). The majority of events were reversible and effectively managed through dose interruptions and reductions, with only less than 1% of patients discontinuing treatment because of QT prolongations (17). Because the delay in cardiac repolarization is unfavorable as it heightens the likelihood of cardiac arrhythmias, particularly torsades de pointes (TdP) (18), recommendations for the management of QTc prolongation with anticancer drugs have been suggested (19). Three mechanisms have been hypothesized to explain the relationship between ribociclib and QTc prolongation. The hypotheses are the consequence of drug–drug interactions (DDIs) due to CYP3A4 inhibition, the human ether‐a‐go‐go‐related gene (hERG) activity inhibition (a marker for cardiotoxicity used in drug development), or modulation of expression of one or more genes such as KCNH2, SCN5A, and SNTA1 (20-23). Previous studies have considered QTc prolongation to be an adverse effect exclusively related to ribociclib (24). Therefore, we performed a systematic review and meta-analysis to identify if QTc prolongation is intrinsic to the class of CDK4/6i or if it is only associated with ribociclib.

## Methods

This systematic review and meta-analysis was conducted and reported in accordance with the Cochrane Handbook of Systematic Reviews of Interventions recommendations and the Preferred Reporting Items for Systematic Reviews and Meta-Analyses (PRISMA) guidelines (25,26). The prospective meta-analysis protocol was registered on PROSPERO (CRD42023440002) on July 3, 2023.

### Search strategy and data extraction

We systematically searched PubMed, Embase, and Cochrane Library in June 2023 for randomized controlled trials (RCTs) comparing CDK4/6i vs no CDK4/6i for patients with breast cancer presenting adverse events. The search strategy used was: (“CDK4/6i” OR “CDK4/6” OR CDK OR “Cyclin Inhibitors” OR “cyclin-dependent kinase” OR ribociclib OR abemaciclib OR Palbociclib OR Ibrance OR Kisqali OR Verzenio) AND (breast OR BC) AND (randomized OR random OR randomized OR RCT). Two authors (BM and PR) independently extracted study characteristics and event rates data from full-text journal articles and pertinent scientific abstracts based on the search strategy, adhering to predefined search criteria and quality assessment. Discrepancies were resolved by consensus among the remaining authors.

### Eligibility criteria

Inclusion in this meta-analysis was restricted to studies that met all the following eligibility criteria: 1) RCTs, 2) comparing patients receiving CDK4/6i with patients not receiving CDK4/6i, 3) enrolling patients with breast cancer, and 4) reporting any outcome of QT prolongation. We excluded studies that 1) were nonrandomized; 2) were secondary analyses of an article included; 3) did not report any outcome of QT prolongation; and 4) were trilaciclib trials, due to the patient population having metastatic TNBC and lack of QTc reports.

### Endpoint, subgroup, and sensitivity analyses

Our primary outcome of interest was any QTc prolongation stratified for each CDK4/6i agent. As per the Common Terminology Criteria for Adverse Events (CTCAE) QTc prolongation is defined as QTc of 450 ms or greater. Grade 1 is QTc of 450 ms to 480 ms, Grade 2 ranges from 481 ms to 500 ms, and Grade 3 is greater than 500 ms or an increase greater than 60 ms from baseline (27). We did not include CTCAE Grade 4 events, such as TdP, polymorphic ventricular tachycardia, or signs/symptoms of serious arrhythmia, because these events were near-nonexistent in the data set. QT correction of choice was Fridericia’s (QTcF) because it is the formula endorsed by the cardio-oncology guidelines (28). Studies that did not specify the method used were presumed to have employed the Bazett formula (QTcB), because it is the default in many automated electrocardiogram (ECG) reporting software packages (29). Between the palbociclib studies (n = 4), only the PALOMA-2 trial specified performing the Fridericia correction; the other 3 studies did not list their correction methods. Subgroup analysis included grade 3 QTc prolongation and a separate analysis of QTc increase from baseline greater than 60 ms, because some studies reported these data separate from grade 3 QTc prolongation.

Given the heterogeneity of the included studies, a sensitivity analysis was conducted for different endocrine therapies, disease stage, and each specific CDK4/6i. We included disease stage as a variable to investigate whether the extent of disease progression could influence the susceptibility to QTc prolongation, given the differential impact of systemic therapy and overall health status at various stages of breast cancer. Furthermore, we conducted a sensitivity analysis without studies using ribociclib to ascertain the collective outcome of CDK4/6i. In addition, we performed a meta-regression analysis for the endpoint of any grade QT-prolongation to assess for any interaction with the mean age of the participants. Last, we collected each individual study 12-lead electrocardiogram schedule regimen.

### Quality assessment

We performed quality assessment using the Cochrane Collaboration’s tool for assessing risk of bias in randomized studies (Rob 2), which allows categorization of each study as low risk, some concerns, or high risk for bias in 5 domains: selection bias, performance bias, detection bias, attrition bias, and reporting bias (30). Two authors (ADM and BMP) performed the risk of bias assessment independently, and disagreements were resolved through consensus. We investigated potential publication bias by employing Egger’s regression test and funnel plot analysis for the outcome of QTc elevation.

### Statistical analysis

All statistical analyses were performed following the intention-to-treat principle whenever available. We pooled relative risks (RRs) with 95% confidence intervals (CIs) for binary endpoints using the Mantel-Haenszel method for all endpoints, subgroups, and sensitivity analyses. A random-effects model was applied to accommodate the demographic and methodological differences observed among the included RCTs, aligning with Cochrane’s recommended methodology (26). Heterogeneity was evaluated through Cochrane Q χ^2^ test and I^2^ statistics with a restricted maximum-likelihood estimator model (31); I^2^ at or greater than 25% and *P* values less than .10 were considered significant for heterogeneity. A *P* value less than .05 was considered statistically significant for treatment effects. We used R software, version 4.3.1 (R Foundation, Vienna, Austria) for statistical analyses.

## Results

### Study selection and baseline characteristics

**Figure 1.**
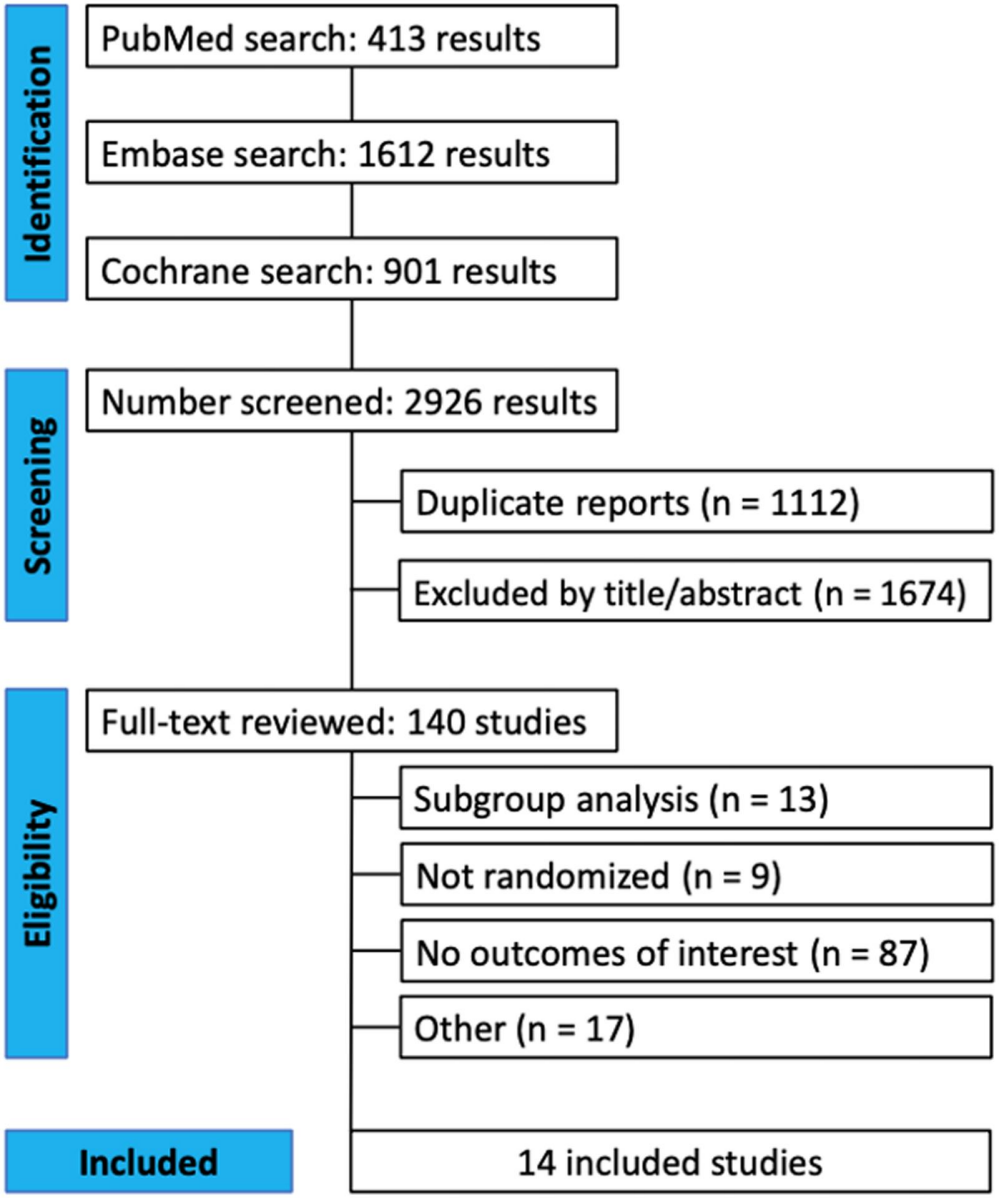
PRISMA flow diagram of study screening and selection.

A comprehensive search yielded a total of 2926 distinct entries, with 1814 studies undergoing screening based on their titles and abstracts after removing duplicates. From this, 140 full-text publications underwent review, as illustrated in Figure 1. Ultimately, 14 RCTs encompassing a total of 16 196 participants were included in the analysis. The characteristics of these trials, including disease stage, combination therapy, follow-up duration, and other pertinent details, are outlined in Table 1 (10,11,32-43). Across the pooled population, the mean age was 57 years, with a mean follow-up duration of 19.2 months. Additionally, the ECOG performance status was 0 and 1 in 64% and 35.7% of patients, respectively.

**Table 1.** Baseline characteristics of included studies

Study, year	Disease stage	Intervention	Control	Phase	Follow up, median	N (Int/Cont)	Age, years, median (range) – Int/Cont	ECOG performance status 0 - Int(%)/Cont(%)	ECOG performance status 1 - Int(%)/Cont(%)	Previous chemo treatment—Int(%)/Cont(%)	Postmenopausal status—Int/Cont, (%)
CORALLEEN, 2019	Early	Ribociclib + Letrozole	Doxorubicin + Cyclophosphamide followed by Paclitaxel	Ph 2	200 days	106 (52/54)	63 (56.5-70.3)/ 64 (58.3-71.8)	NA	NA	NA	100/100
DAWNA—1, 2021	Advanced	Dalpiciclib + Fulvestrant	Placebo + Fulvestrant.	Ph 3	10.7 mo/ 10.6 mo^a^	361 (241/120)	50.7 (45.3-59.3)/ 52.4 (45.5-60.6)	116 (48.1)/ 46 (38.3)	125 (51.9)/ 74 (61.7)	65 (27.0)/ 42 (35.0)	56/55
DAWNA—2, 2023	Advanced	Dalpiciclib + Letrozole or Anastrozole	Placebo + Letrozole or Anastrozole	Ph 3	21.6 mo	456 (303/153)	54 (47-63)/ 57 (46–63)	141 (47)/ 69 (45)	161 (53)/ 84 (55)	161 (53)/ 79 (52)	60/65
MAINTAIN, 2023	Advanced	Ribociclib + Fulvestrant or Exemestane	Placebo + Fulvestrant or Exemestane	Ph 2	18.2 mo	119 (59/60)	59 (51.5-65)/ 55 (48-67)	38 (64.4)/ 40 (66.7)	21 (35.6)/ 20 (33.3)	7 (11.9)/ 4 (6.7)	NA^c^
MONALEESA-1, 2016^d^	Early	Ribociclib + Letrozole	Letrozole	Ph 2	15 days	14 (6/4/4)	65 (51-78)/ 70 (66-75)/ 57 (51-63)	4 (67)/ 3 (75) 4 (100)	2 (33)/ 1 (25)/ 0	NA	100/100/100
MONALEESA-2, 2022	Advanced	Ribociclib + Letrozole	Placebo + Letrozole	Ph 3	15.3 mo	668 (334/334)	62 (23-91)/ 63 (29–88)	205 (61.4)/ 202 (60.5)	129 (38.6)/ 132 (39.5)	146 (43.7)/ 145 (43.4)	100/100
MONALEESA-3, 2021	Advanced	Ribociclib + Fulvestrant	Placebo + Fulvestrant	Ph 3	39.4 mo	726 (484/242)	63 (31-89)/ 63 (34–86)	310 (64)/ 158 (65.3)	173 (35.7)/ 83 (34.3)	274 (56.6)/ 131 (54.1)	100/100
MONALEESA-7, 2022	Advanced	Ribociclib + Tamoxifen or Letrozole or Anastrozole	Tamoxifen or Letrozole or Anastrozole	Ph 3	19.2 mo	672 (335/337)	43 (25-58)/ 45 (29–58)	245 (73)/ 255 (76)	87 (26)/ 78 (23)	185 (55)/ 185 (55)/	0/0
MONARCH 2, 2019	Advanced	Abemaciclib + Fulvestrant	Placebo + Fulvestrant	Ph 3	47.7 mo	669 (446/223)	59 (32-91)/ 62 (32-87)	264 (59.2)/ 136 (61.0)	176 (39.5)/ 87 (39.0)	267 (59.9)/ 134 (60.1)	83.2/80.7
NATALEE, 2023	Early	Ribociclib + Letrozole or Anastrozole	Letrozole or Anastrozole	Ph 3	34 mo	5101 (2549/2552)	52 (24-90)/ 52 (24-89)	2106 (83)/ 2132 (84)	440 (17)/ 418 (16)	2249 (88)/ 2245 (88)	56/56
PALLAS, 2021	Early	Palbociclib + Tamoxifen or AI	Tamoxifen or AI	Ph 3	31 mo	5761 (2884/2877)	52 (25.0-90.0)/ 52.0 (22.0-85.0)	2403 (83.3)/ 2404 (83.6)	478 (16.6)/ 470 (16.3)	2384 (82.7)/ 2370 (82.4)^b^	54.2/53.3
PALOMA-2, 2016	Advanced	Palbociclib + Letrozole	Placebo + Letrozole	Ph 3	23 mo	666 (444/222)	62 (30-89)/ 61 (28–88)	257 (57.9)/ 102 (45.9)	178 (40.1)/ 117 (52.7)	213 (48.0)/ 109 (49.1)	100/100
PALOMA-3, 2016	Advanced	Palbociclib + Fulvestrant	Placebo + Fulvestrant.	Ph 3	8.9 mo	521 (347/174)	57 (30-88)/ 56 (29–80)	206 (59)/ 116 (67)	141 (41)/ 58 (33)	252 (73)/ 138 (79.3)	79/79
PALOMA—4, 2022	Advanced	Palbociclib + Letrozole	Placebo + Letrozole	Ph 3	52.8 mo	340 (169/171)	54.0 (31-70)/ 54.0 (29-70)	84 (49.7)/ 81 (47.4)	85 (50.3)/ 90 (52.6)	126 (74.6)/ 129 (75.4)	100/100

Baseline characteristics of included studies: Data are n (%) unless otherwise indicated; ECOG = Eastern Cooperative Oncology Group performance status; mo = months; NA = not applicable.

a Median follow-up for intervention and control group, respectively.

b One patient had unknown prior chemotherapy data.

c Study reported as inclusion criteria: postmenopausal status or receiving ovarian ablation with a GnRH agonist such as goserelin.

d Data of MONALEESA-1 is presented in 3 arms: (Arm 1) Letrozole 2.5 mg/day, (Arm 2) Ribociclib 400 mg/day + letrozole 2.5 mg/day, (Arm 3) Ribociclib 600 mg/day + letrozole 2.5 mg/day.

### Pooled analysis of all studies

#### All grades QT elevation

**Figure 2.**
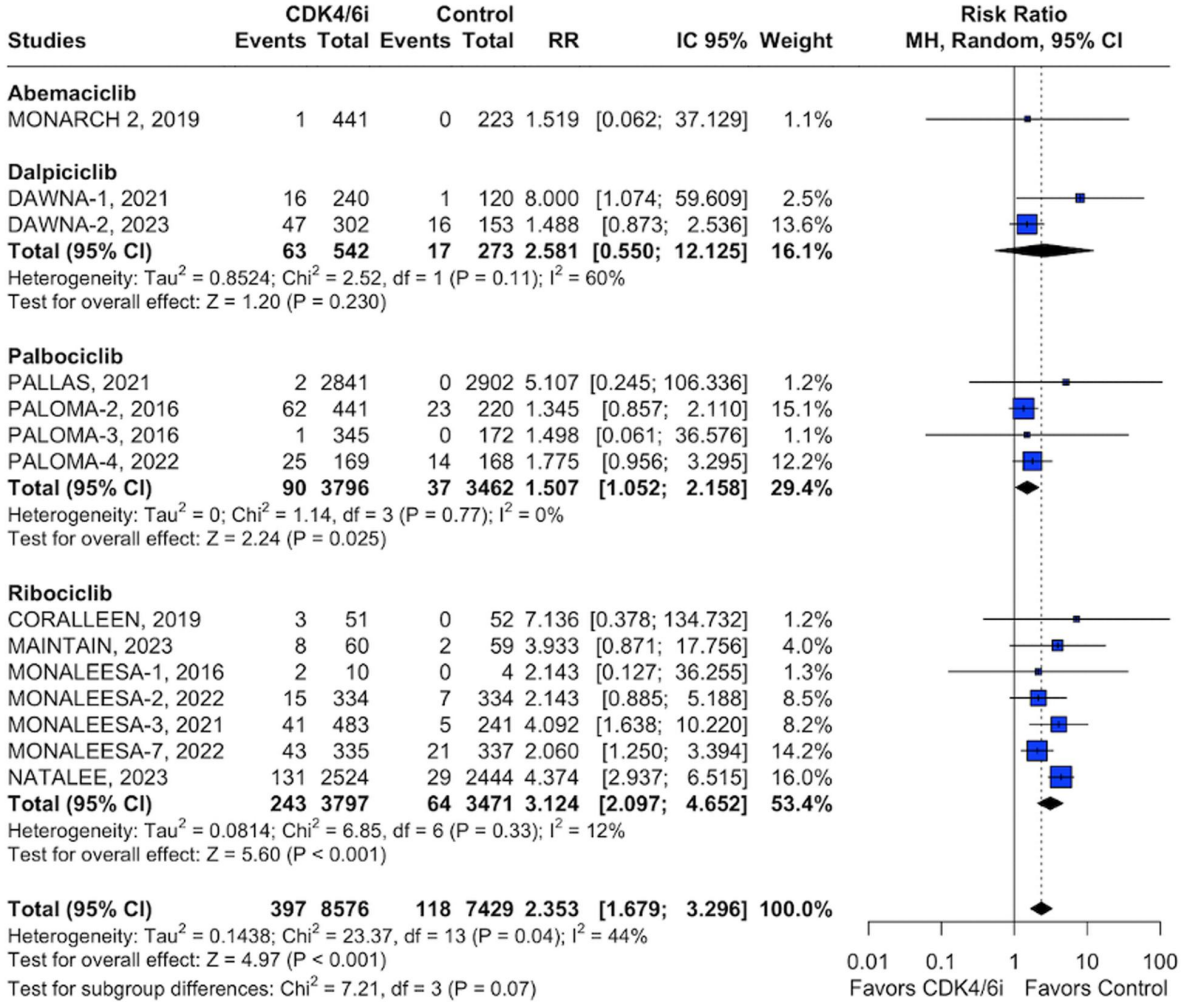
Pooled analysis of all studies for all grades QT elevation.

Of 8576 patients included in the quantitative analysis of the intervention group, 397 QTc prolongation events were recorded. Ribociclib exhibited the highest incidence of QTc prolongation events (n = 243) with a relative risk of 3.12 (95% CI = 2.09 to 4.65, *P* < .001; Figure 2). Palbociclib, the subgroup with the second highest number of QTc prolongation events (n = 90), showed a relative risk of 1.50 (95% CI = 1.05 to 2.15, *P* = .025; Figure 2). Additional analysis for dalpiciclib (n = 63) yielded a relative risk of 2.58 (95% CI = 0.55 to 12.12, *P* = .230). Similarly, analysis for abemaciclib (n = 1) was constrained by the scarcity of available data (Figure 2).

### Subgroup analyses

#### Grade 3 QT prolongation

Regarding grade 3 QTc prolongation events, ribociclib maintained the highest number of reported events (n = 49) and demonstrated statistical significance with a relative risk of 2.17 (95% CI = 1.28 to 3.67, *P* = .004). Palbociclib, with 5 events, showed a relative risk of 1.49 (95% CI = 0.37 to 6.03, *P* = .57), and dalpiciclib (n = 4) had a relative risk of 1.42 (95% CI = 0.21 to 9.33, *P* = .23; Supplementary Figure 1, available online).

#### QT increase >60 ms

The analysis for a QT increase greater than 60 ms was feasible only for ribociclib and palbociclib. This analysis revealed a significantly higher risk in the intervention arm when compared with control (RR = 5.84, 95% CI = 2.91 to 11.71, *P* < .001; Supplementary Figure 2, available online).

#### Sensitivity analyses

The findings of all sensitivity analyses remained consistent with the primary analysis. Tamoxifen sensitive analyses were limited due to a lack of available data. Pooled analysis for this hormonal treatment involved only 2 trials, each one with different CDK4/6i. MONALESSA-7 study (37), combined TAM with ribociclib and PALLAS trial (40), which combined TAM with palbociclib, reported together a relative risk of 2.57 (95% CI = 1.07 to 6.14, *P* = .033) (Supplementary Figure 3, available online). In the Aromatase Inhibitors (AI) analysis, a subgroup was made for studies using only letrozole and for studies reporting letrozole or anastrozole. Patients using exclusively letrozole presented the lowest QT prolongation association (RR = 1.60, 95% CI = 1.14 to 2.23; *P* = .006) when compared with studies reporting letrozole or anastrozole population (RR = 3.79, 95% CI = 1.03 to 13.9, *P* = .044) (Supplementary Figure 4, available online). The combined analysis for all AI presented a RR of 2.25 (95% CI = 1.42 to 3.59, *P* < .001). For fulvestrant, the relative risk for QT prolongation was 4.02 (95% CI = 1.84 to 8.78; *P* < .0001) (Supplementary Figure 5, available online). Additionally, for the stage disease subanalysis, no substantial difference was observed from the overall results. In the sensitivity analysis, studies comprising an advanced-stage population had a relative risk of 1.83 (95% CI = 1.45 to 2.31, *P* < .001) (Supplementary Figure 7, available online), whereas studies with early-stage populations showed a relative risk of 4.36 (95% CI = 2.96 to 6.43, *P* < .001) (Supplementary Figure 6, available online). Last, for the analysis without ribociclib, the forest plot demonstrated a relative risk of 1.55 (95% CI = 1.16 to 2.08, *P* = .003), (Supplementary Figure 8, available online). However, when analyzing studies without ribociclib and palbociclib, no statistically significant results were found, suggesting that palbociclib was the drug driving the significant results in the previous analysis (Supplementary Figure 9, available online). A meta-regression analysis suggested no significant interaction between all grade QT-prolongation and the covariate of mean patient age (Supplementary Figure 10, available online).

In Supplementary Table 2 (available online), the 12-lead electrocardiogram schedule for each RCT demonstrated a generally consistent approach to QT monitoring, although certain palbociclib studies featured fewer assessment instances. Between palbociclib studies, both PALLAS and PALOMA-3 trials, each with only 2 defined ECG schedules, were associated with fewer QTc prolongation events (3 out of 6.260 in the intervention and controlled group) when compared with the other 2 palbociclib studies PALOMA-2 and PALOMA-4 (124 out of 998) (Figure 2).

#### Quality assessment

Quality assessment of each RCT is presented in Supplementary Figures 11 and 12 (available online). Rob 2 identified 1 study at high risk of bias (30). All other studies carried a low risk of bias. Egger’s regression test suggested no evidence of publication bias (*P* = .59; Supplementary Figure 13, available online).

## Discussion

In this systematic review and meta-analysis of RCTs, we assessed the relative risk of CDK4/6i-induced QTc prolongation. Our main findings were as follows: (1) Ribociclib was associated with the most pronounced increase in QT interval, (2) there was an association between palbociclib and QT interval elevation, (3) dalpiciclib and abemaciclib when evaluated alone were not associated with QTc prolongation, and (4) Grade 3 and increase of greater than 60 ms subgroup analyses results sustained a significant overall effect of QT prolongation in the CDK4/6i arm compared with the placebo arm. It is important to note that within these last 2 subgroup analyses, ribociclib was predominant over the other drugs, introducing bias to the overall effect. Furthermore, the sensitivity analysis excluding ribociclib studies was predominantly influenced by palbociclib trials, showing a statistically significant outcome for QT prolongation. This heavy weighting made it difficult to draw firm conclusions regarding the effects of abemaciclib and dalpiciclib. These results still support the fact that an increase in the QT interval is not a CDK4/6i class effect, as our sensitivity analysis with dalpiciclib and abemaciclib studies demonstrated no difference between CDK4/6i and placebo. In summary, our results indicate that QT prolongation is a direct drug effect of both ribociclib and palbociclib, with the effect of palbociclib at less than 50% that of ribociclib. However, the association between this cardiac adverse event and dalpiciclib and abemaciclib requires further data for clarification.

In addition, disease stage sensitivity analysis demonstrated a higher correlation between CDK4/6i inducing QT prolongation events for early-stage populations instead of advanced-stage patients. Last, for the endocrine therapy analysis, despite the limitations, the combined TAM analysis from the PALLAS and MONALESSA-7 trials (37,40) revealed a significantly higher risk of QT prolongation in the CDK4/6i group. The MONALESSA-7 study led to the FDA recommending against the combination of ribociclib and TAM (15). Guided majorly by the MONALESSA-3 study (36), pooled analysis for fulvestrant reported the highest QT prolongation risk among all endocrine therapies. Regarding AI analysis, studies with patients receiving letrozole or anastrozole alongside CDK4/6i reported significantly more QT prolongation adverse events when compared with studies involving only letrozole. However, no definitive conclusions can be drawn from this observation.

Reasons for the more pronounced effect of CDK4/6i on QT interval may be multifactorial. An aspect contributing to the observed variations in QT interval prolongation may stem from the interplay between prior lines of treatment, patient age, and the concurrent selection of endocrine therapies. Our data were limited to this analysis and could not observe a relationship between patients with advanced disease to a higher incidence of QT elevation than patients with early disease. This might be a result of studies’ substantial heterogeneity in terms of sample size, potentially introducing bias to align with the outcomes of the largest study within our analysis. Patients who used concomitant fulvestrant compared with AIs or TAM had a similar rate of QT prolongation, but a comparison between AIs and TAM seems to influence the number of events. Two previously reported studies, PALLAS and MONALEESA-7, featured a subset of patients using TAM (37,40). Intriguingly, only 1 (MONALEESA-7) reported the number of events in the TAM group separately, revealing more QT elevation events in the cohort treated with TAM. Additionally, as noted by Richardson and colleagues (29), the QTcB formula overestimates QTc values compared with QTcF. Based on this premise, if all reports that did not specify the QTc formula used QTcB calculations, the results would represent a “worst-case” scenario for QTc prolongation compared with findings where QTcF were consistently applied.

In contrast to previous investigations asserting the impact of palbociclib on QT intervals, our study reveals an association between palbociclib administration and QT interval prolongation, even when analyzed in isolation. It is essential to acknowledge a significant discrepancy in the documented occurrences of QT interval abnormalities among the palbociclib studies, potentially introducing bias into our analysis. Particularly, the studies with the most notable percentage of QT prolongations, PALOMA-2 and PALOMA-4, had consistent findings of around 14% of patients experiencing QT prolongations, with most of these events being grade 1 or 2 (PALOMA-2 and 1) (41,43).

It is crucial to consider the potential role of CYP450 inhibition as another contributing factor to QTc prolongation associated with the use of CDK4/6 inhibitors when combined with other QT-prolonging agents that are also major CYP450 substrates. Earlier investigations have demonstrated that ribociclib exerts inhibitory effects on the activities of 4 CYP isoforms—namely, CYP1A2, CYP3A4, CYP3A5, and CYP2C9 (15). Specifically, ribociclib is a moderate CYP3A4 inhibitor. As a result, ribociclib can introduce significant drug–drug interactions, including the relative increase in QT prolongation events observed with the combination of ribociclib and tamoxifen in MONALEESA-7 (incidence = 16%, 14 of 87) compared with ribociclib and aromatase inhibitor combination (incidence = 7%, 18 of 245). Abemaciclib has no clinically meaningful effect on CYP1A2, CYP2C9, CYP2D6, and CYP3A4 substrates (44). On the other hand, palbociclib was shown to weakly inhibit CYP3A4 enzyme, making it unlikely to induce clinically significant interactions with CYP3A4 substrates (45).

The hERG gene is intricately linked to the Rapid Delayed Rectifier K+ current (IKr). The observed variations in the cardiotoxic effects of certain drugs may be attributed to their influence on hERG activity. Drugs that inhibit the hERG-encoded potassium channel have been identified as potential contributors to QT interval prolongation. In the preclinical evaluation of a drug, the widely accepted marker for cardiotoxicity is the assessment of its interaction with hERG, typically through methods such as the tail current assay. The cardiac safety of a drug is assessed by evaluating its IC50 value, which represents the concentration at which 50% of hERG current is blocked, and the Cmax free, indicative of the maximum drug concentration unbound to plasma proteins. The ratio between IC50 (hERG) and Cmax free serves as a critical parameter for predicting cardiac safety, with a recommended safety margin of at least 30-fold or higher for hERG. The IC50 (hERG)/Cmax free ratio for palbociclib ranges from about 51 to 82, whereas for ribociclib it ranges from about 17 to 44 (21,22). In vitro studies of abemaciclib and its major active metabolites M2 and M20 have not demonstrated blockade of the current produced by the hERG potassium channel expressed in mammalian cells (unpublished data). Results further support that abemaciclib has no significant effect on QTc increase (46).

Additionally, the genetic dimension comes into play, with microarray analysis revealing differential expression of KCNH2, SCN5A, and SNTA1—genes associated with long QT syndrome—when cells are treated with ribociclib. This includes the downregulation of KCNH2 and the upregulation of SCN5A and SNTA1 (22,47).

### Study implications

To the best of our knowledge, this marks the first meta-analysis specifically examining the association between CDK4/6 inhibitors and QT prolongation in breast cancer patients. Our findings indicate a consistent link between QT prolongation and the use of CDK4/6 inhibitors. However, evidence supporting a correlation with more severe and clinically important adverse effects, such as TdP or Syncope, remains limited. Notably, none of the studies included in this review reported instances of TdP. Moreover, in cases where QT prolongation occurred, it consistently improved with either dose reduction or cessation of the CDK4/6 inhibitor.

The overall effect of ribociclib on QT prolongation aligns with previous reports (14). Interestingly, pooled data from studies on palbociclib revealed an association with QT prolongation, a finding not previously identified (24,48-50). A study published in 2018 presents opposing data, suggesting that palbociclib, when co-administered with letrozole, resulted in a QTc increase of less than 10 ms (49). According to the ICH E14 guideline, the threshold level of regulatory concern for QTc prolongation is that the upper bound of the one-sided 95% CI around the largest time-matched mean effect on QTc is less than 10 ms, and in the context of oncology drugs, a threshold level of less than 20 ms is widely accepted (51).

Further research should explore the specific mechanisms underlying QT prolongation and evaluate its clinical implications for the safe and effective use of CDK4/6 inhibitors in breast cancer treatment.

### Study limitations

Although our study provides valuable insights, it is important to acknowledge certain limitations. First, the exploration of a potential association between TAM and CDK4/6 inhibitors was impeded by limited data on TAM as well as for other medications intrinsically linked to QTc prolongation. This can be related to MONALEESA-7, which showed an increase in QTc prolongation that precludes the use of tamoxifen with ribociclib. This investigation is of particular interest because ribociclib inhibits CYP3A and tamoxifen is a major CYP3A4 substrate. Exploring the association of ribociclib with TAM, which is also metabolized by CYP3A and linked to QT prolongation, could elucidate the mechanism of increase in QT prolongation observed with the combination. Additionally, because there was not an available median age specifically for the QT prolongation population, the estimate of weighted average ages for the meta-regression analysis had to be calculated from the median age of all patients from each individual study, representative in some cases of a broader number of patients, some of which have not been evaluated for QT prolongation. A table showing the differences between those numbers of patients can be seen in the Supplementary Material (Supplementary Table 1, available online). Second, some of the included studies did not specify whether QT prolongation on ECGs was corrected. Consequently, our analysis compared prolonged QT intervals with studies that did specify correction, introducing a potential source of variability. Third, the evaluation of individual patients’ concomitant use of other drugs and herbal products or other supplements during treatment was not possible. This limitation is notable, because drugs known to be associated with QT prolongation could introduce bias into the analysis. Furthermore, our analysis of patients whose QTc elevated more than 60 ms from baseline was constrained by data limitations. This subgroup, composed of only 4 studies (3 on ribociclib and 1 on palbociclib), made it impossible to subcategorize by drugs and draw conclusions about the effects of drugs other than ribociclib. Last, despite no constant differences seen in the frequency of ECG monitoring, QTc is not routinely monitored in some palbociclib studies, and the paucity of data pertaining to abemaciclib potentially could lead to inherited bias.

In patients with breast cancer, ribociclib had the greatest impact on QT interval. Palbociclib exhibited an association with QTc prolongation. However, there were no significant differences in the incidence of TdP. These results provide reassurance regarding the overall safety profile of this drug class. Oncologists should also be aware of the formula used to calculate QTc intervals, recognizing that the Bazett formula may be less appropriate for clinical decision-making than the QTcF formula. Further investigation is warranted to better understand the mechanisms and clinical implications of CDK4/6i induced QTc prolongation.

## Supplementary Material

pkae078_Supplementary_Data

## Data Availability

The data underlying this article are available in the article and in its online supplementary material.
