# Supplementary material for: QTc prolongation across CDK4/6 inhibitors: a systematic review and meta-analysis of randomized controlled trials
Source: JNCI Cancer Spectr. 2024 Sep 10;8(5):pkae078. doi: 10.1093/jncics/pkae078 (PMC11460542; doi:10.1093/jncics/pkae078)

**Supplementary Material**

Bruno Murad, MD^#1^, Pedro C. A. Reis^#2^, Alice Deberaldini Marinho^3^, Ana Carolina Marin Comini, MD^4^, Débora Pinheiro Xavier, MD^5^, Beatriz Mella Soares Pessoa, MD^6^, Farah Raheem, Pharm.D., R.Ph., BCOP^7^, Brenda Ernst, MD^7^, Lida A. Mina, MD^7^, Felipe Batalini, MD,^7^

*^1^ MD, Faculdade de Medicina de Barbacena (FUNJOB), Minas Gerais, Brazil*

*^2^ Medical Student, Universidade Federal do Rio de Janeiro, Rio de Janeiro, Brazil*

*^3^ Medical Student, Universidade Federal do Estado do Rio de Janeiro (UNIRIO), Rio de Janeiro, Brazil*

*^4^ MD, A. C. Camargo Cancer Center, São Paulo, Brazil*

*^5^ MD, Universidade Federal do Pará (UFPA), Pará, Brazil*

*^6^ University of Connecticut, Farmington, CT, USA*

*^7^ Mayo Clinic, Phoenix, AZ, USA*

*^#^* ***Contributed equally.***

**Table of Contents**

[Supplementary Figure 1**.** Subgroup analysis for GRADE III QTc 3](#_Toc162415867)

[Supplementary Figure 2. Subgroup analysis for QTc increase >60ms 4](#_Toc162415868)

[Supplementary Figure 3. Sensitivity analysis for Tamoxifen 5](#_Toc162415869)

[Supplementary Figure 4. Sensitivity analysis for Aromatase Inhibitors 6](#_Toc162415870)

[Supplementary Figure 5. Sensitivity analysis for Fulvestrant 8](#_Toc162415871)

[Supplementary Figure 6. Sensitivity analysis for Early Stage 9](#_Toc162415872)

[Supplementary Figure 7. Sensitivity analysis for Advanced Stage 10](#_Toc162415873)

[Supplementary Figure 8. Sensitivity analysis without Ribociclib 11](#_Toc162415874)

[Supplementary Figure 9. Sensitivity analysis without Ribociclib and Palbociclib 12](#_Toc162415875)

[Supplementary Figure 10. Metaregression assessing the impact of age on all grade QT-prolongation 13](#_Toc162415876)

[Supplementary Table 1. Metaregression Data 14](#_Toc162415884)

[Supplementary Table 2. 12-Lead electrocardiogram schedule regimen 15](#_Toc165890054)

[Supplementary Figure 11. Critical appraisal of individual studies 16](#_Toc162415885)

[Supplementary Figure 12. Summary of risk of bias 16](#_Toc162415886)

[Supplementary Figure 13. Funnel plot analysis and Egger’s regression test 17](#_Toc162415887)

Supplementary Figure 1**.** Subgroup analysis for GRADE III QTc.


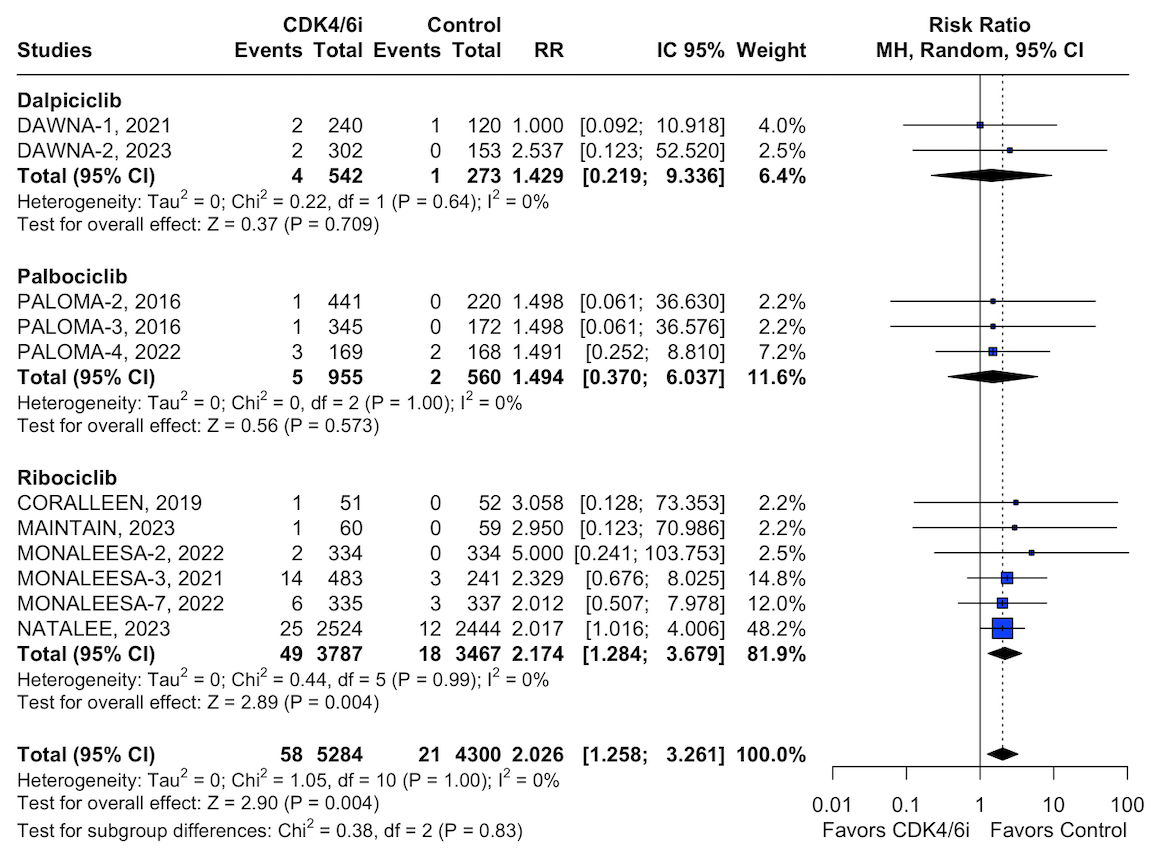


Supplementary Figure 2**.** Subgroup analysis for QTc increase >60ms.

**
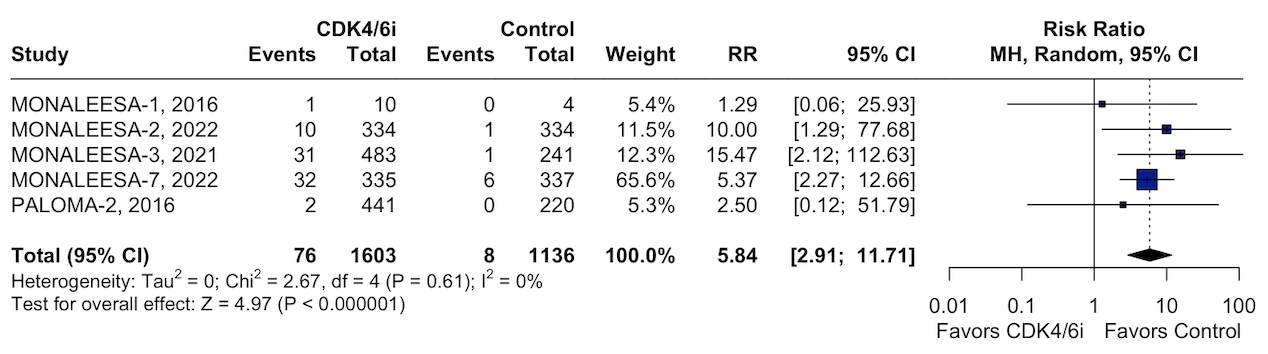
**

Supplementary Figure 3**.** Sensitivity analysis for Tamoxifen

**
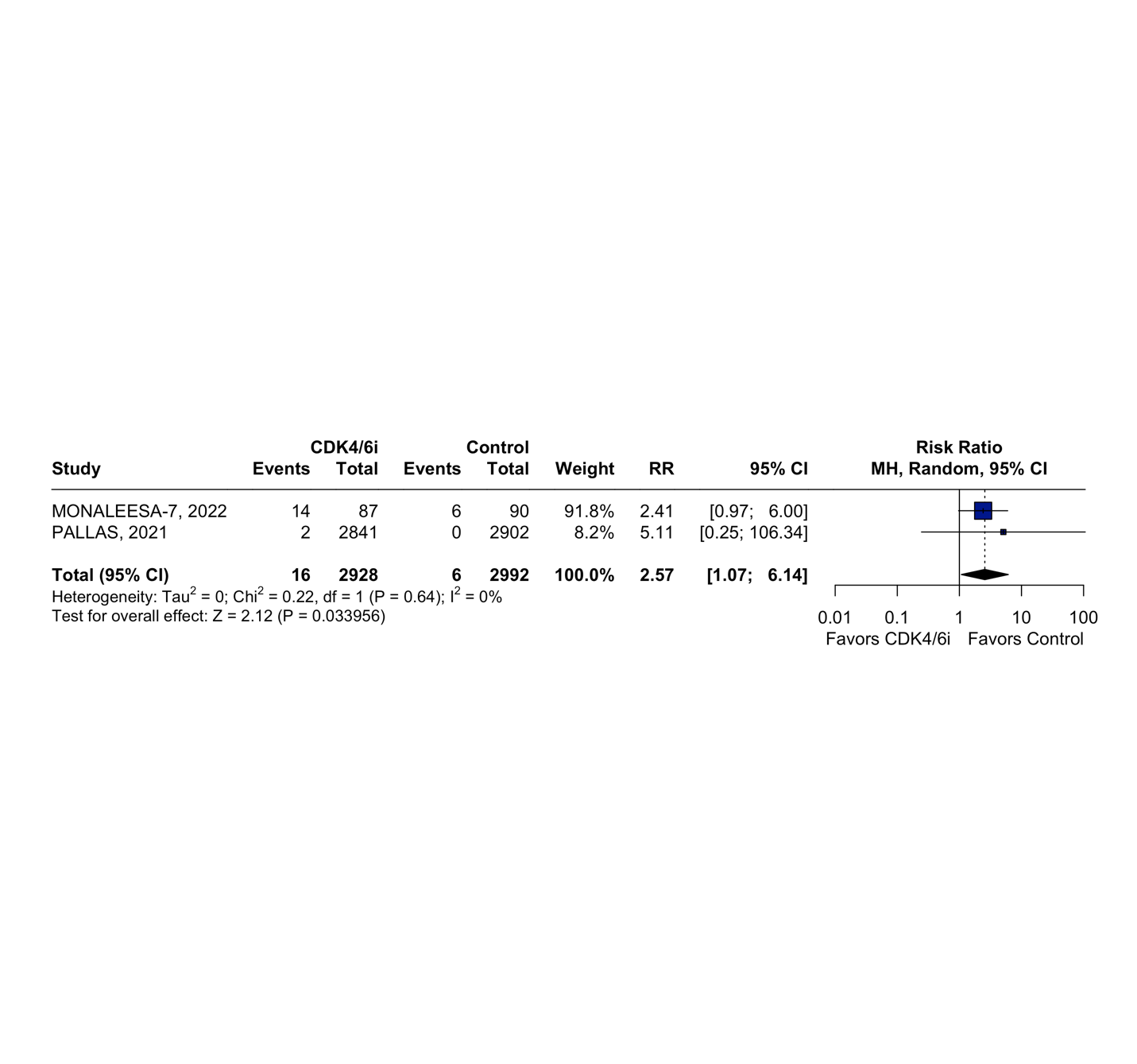
**

Supplementary Figure 4**.** Sensitivity analysis for Aromatase Inhibitors.

**
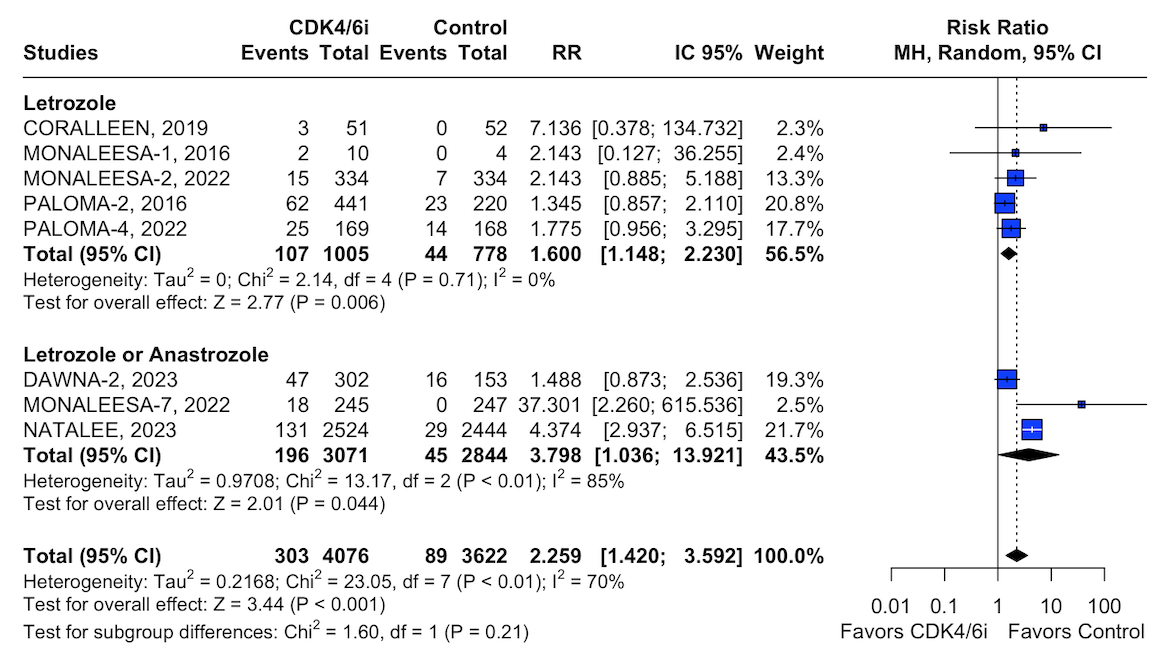
**

Supplementary Figure 5**.** Sensitivity analysis for Fulvestrant.


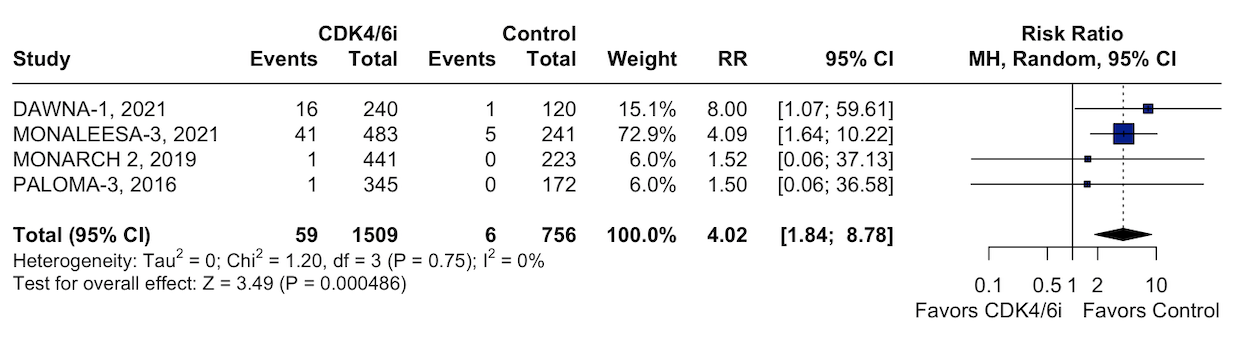


Supplementary Figure 6**.** Sensitivity analysis for Advanced Stage.


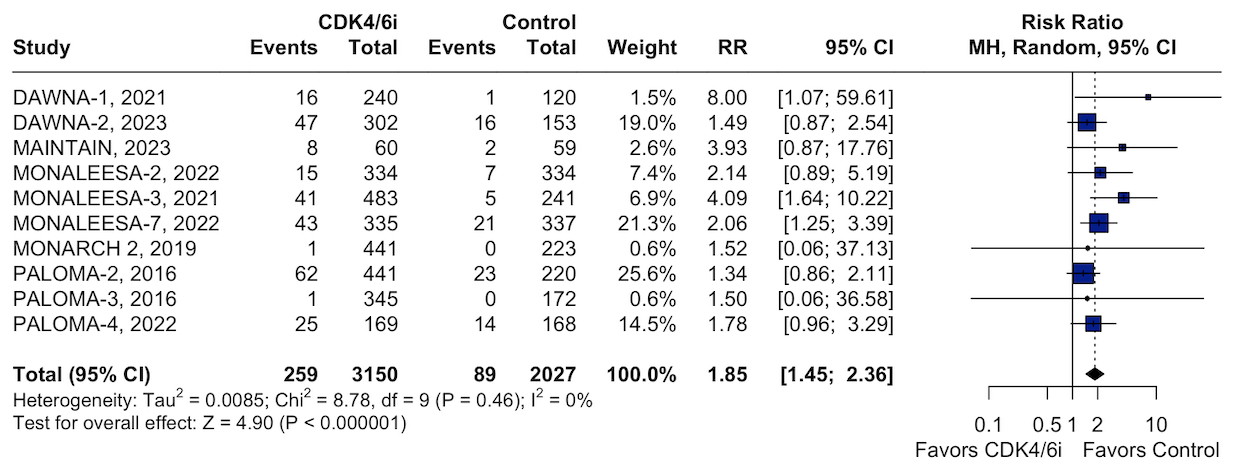


Supplementary Figure 7**.** Sensitivity analysis for Early Stage.


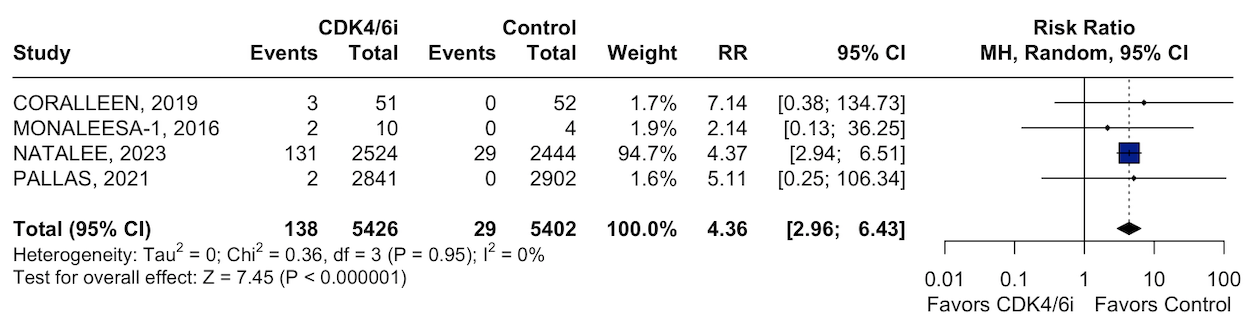


Supplementary Figure 8**.** Sensitivity analysis without Ribociclib.


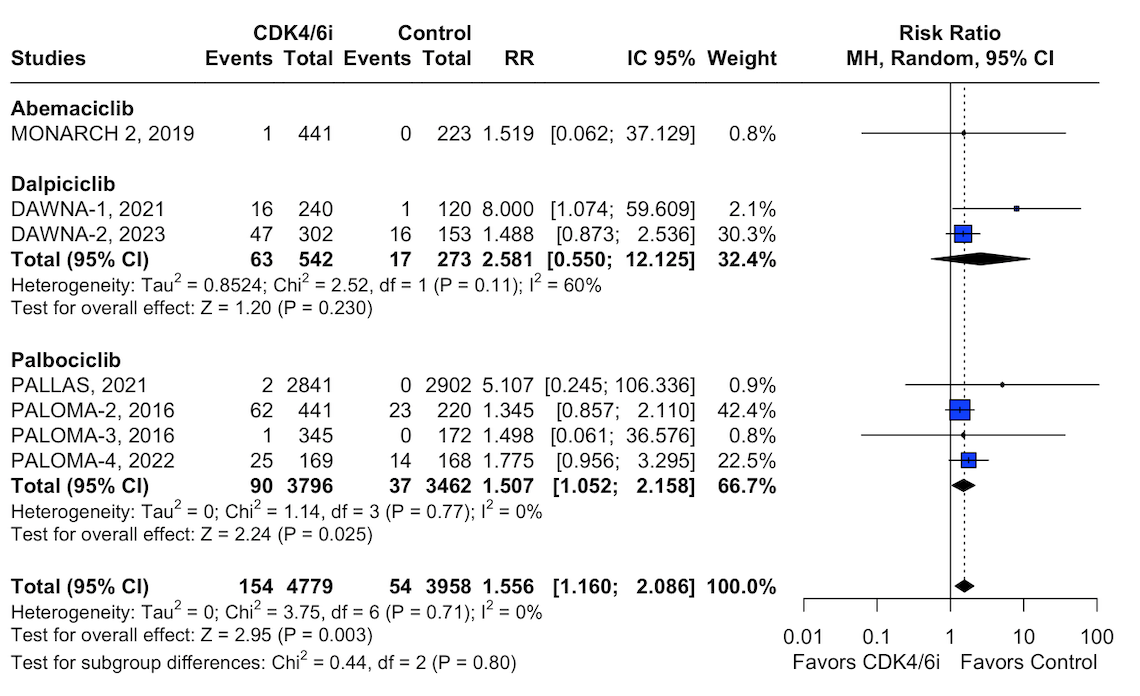


Supplementary Figure 9**.** Sensitivity analysis without Ribociclib and Palbociclib.


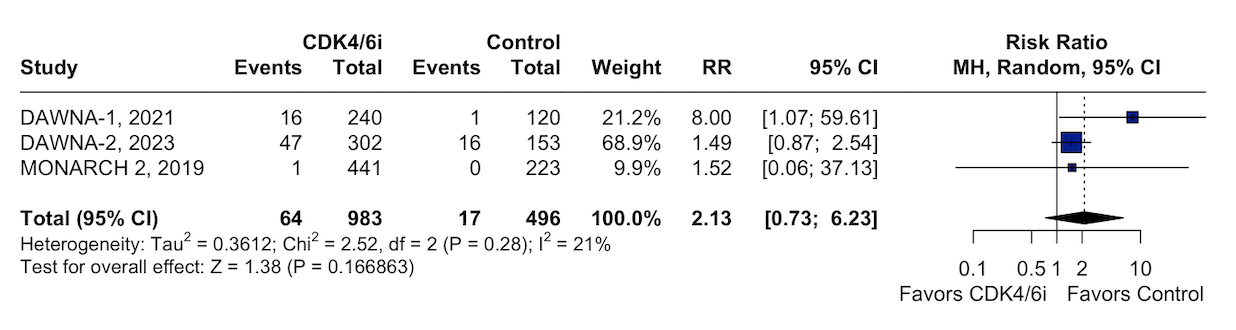


Supplementary Figure 10. Metaregression assessing the impact of age on all grade QT-prolongation.

**
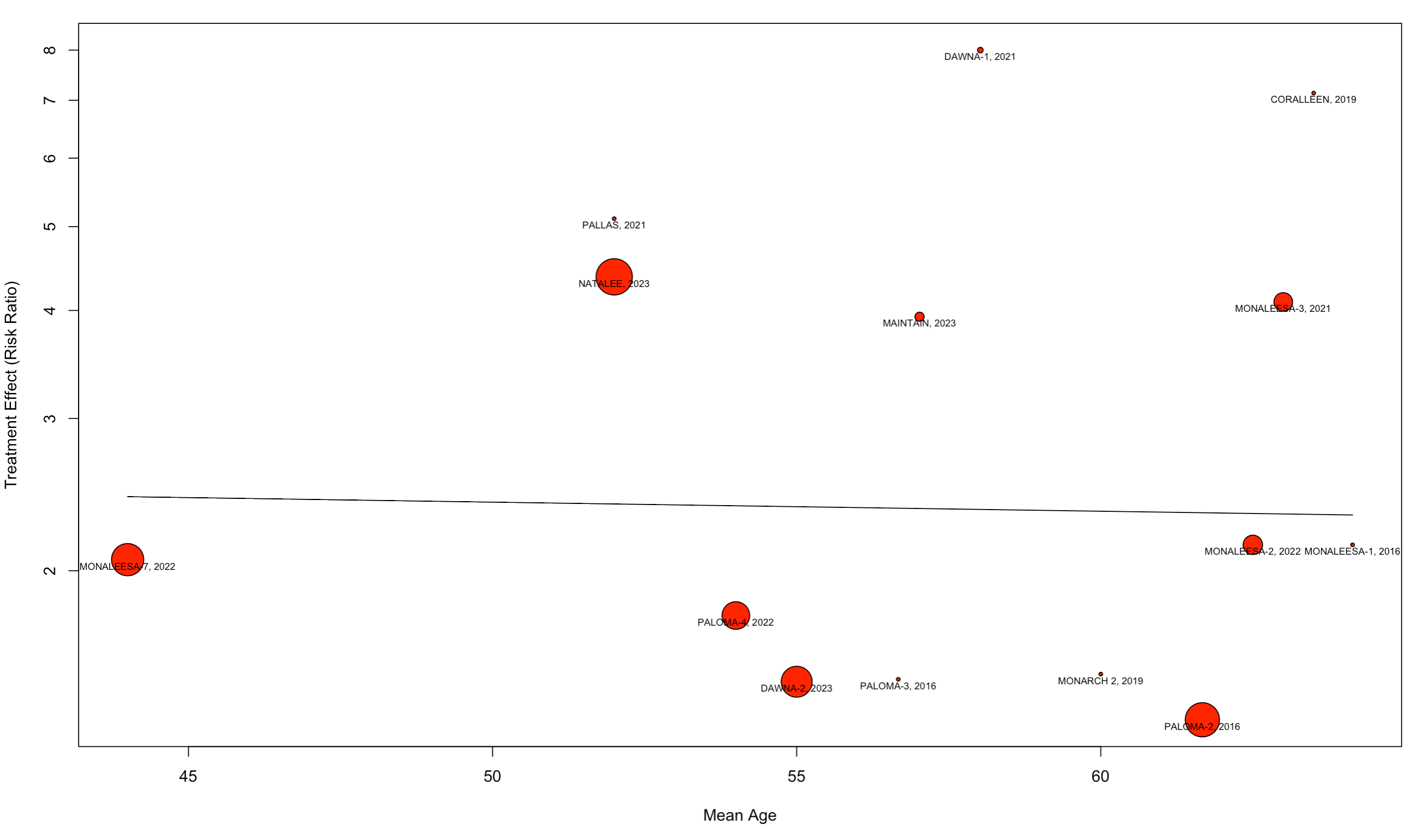
**

|  | Effect Estimate | p-value | I^2^ | Test for Residual Heterogeneity |
| --- | --- | --- | --- | --- |
| Intercept | 0.9983 | 0.5457 | 48.69% | p=0.0364 |
| Age | -0.0024 | 0.9340 |  |  |

Supplementary Table 1. Metaregression data

| Study | Total Population in which median age was calculated – Int/Cont | Number of patients that had QT-prolongation evaluated – Int/Cont |
| --- | --- | --- |
| CORALLEEN | 52/54 | 51/52 |
| DAWNA - 1 | 241/120 | 240/120 |
| DAWNA - 2 | 303/153 | 302/153 |
| MAINTAIN | 60/59 | 60/59 |
| MONALEESA-1 | 6 - 4/4 | 6 - 4/4 |
| MONALEESA-2 | 334/334 | 334/334 |
| MONALEESA-3 | 484/242 | 483/241 |
| MONALEESA-7 | 335/337 | 335/337 |
| MONARCH 2 | 446/223 | 441/223 |
| NATALEE | 2549/2552 | 2524/2444 |
| PALLAS | 2884/2877 | 2841/2902 |
| PALOMA-2 | 444/222 | 441/220 |
| PALOMA-3 | 347/174 | 345/172 |
| PALOMA-4 | 169/171 | 169/168 |

Supplementary Table 2. 12-Lead electrocardiogram schedule regimen

| Study | CDK4/6i arm | Control Arm |
| --- | --- | --- |
| CORALLEEN | Screening /// W3 - C1D15 /// W5 C2D1 /// W9 C3D1 /// W17 C5D1 /// W21 C6D1 /// W7 C2D15 /// W13 C4D1 AND Pre-surgery or ET Visit | -Screening (-28 to -1 days) /// Pre-surgery or ET Visit  -Doxorubicine and cyclophosphamide: W4 C2D1 /// W7 C3D1 /// W10 C4D1 ///  -Paclitaxel: W13 C1D1 /// W16 C4D1 /// W19 C7D1 /// W22 C10D1 |
| DAWNA - 1 | Screening Period D -7 to D -1 /// C1D1 ± 3 /// C2 and beyond D1 ± 3 /// End-of- Treatment Visit (14 Days after the Last Dose /// Safety (28 ± 7 Days after the Last Dose) /// (Follow-Up Period) - As needed | |
| DAWNA - 2 | Screening Period D -7 to D -1 /// C1D1 ± 3 /// C2 and Beyond - D1 ± 3 /// End-of-Treatment Visit | |
| MAINTAIN | C1D1 /// C1D15 /// C2D1 and C4D1. | |
| MONALEESA-1 | 72 h prior to randomization /// D1, 8, and 14 at the following time points: pre-dose and 2, 4, and 6 h after treatment dose. | |
| MONALEESA-2 | Screening /// C1D15 /// C2D1 and C3D1. After a protocol amendment, additional ECG assessments were carried out on: C4D1 /// C5D1 /// C6D1 /// C7D1 /// C8D1 /// C9D1 and on day 1 of subsequent cycles in patients with a mean QTcF of 481 msec at any time before cycle 10 | |
| MONALEESA-3 | Screening /// C1D15 /// C2D1 /// C2D15 /// C3D1 /// C4D1 /// C5D1 /// C6D1 /// at end of treatment and as clinically indicated. In patients with a QTcF ≥ 481 ms at any time before cycle 7, additional ECGs were performed pre-dose on D1 of subsequent cycles and post-dose every third cycle. | |
| MONALEESA-7 | C1D1 /// C1D15 /// C2D1 /// C3D15 /// D1 of all subsequent C and at the end of treatment.  After a protocol amendment on April 28, 2015, an ECG assessment was also done on C3D1, and ECG assessments were only required after C6 if a QTcF of at least 481 ms had been recorded any time before C7. After another protocol amendment on Feb 17, 2016, triplicate 12-lead ECGs were used for all assessments for consistency across clinical trials of ribociclib. | |
| MONARCH 2 | Baseline (Day -14 to Day -1) //// C1D1 //// C1D15 //// C4D1 //// 30-Day Follow-Up | |
| NATALEE | Screening (-28 to -1) /// Cycle 1 Day 15 /// Cycle 2 Day 1 /// Cycle 2 Day 15 /// Cycle 3 Day 1 /// Cycle 4 Day 1 /// Cycle 5 Day 1 /// Cycle 6 Day 1 /// Cycle 7 and every 3rd cycle until 36 months from randomization (Patients with QTcF ≥ 481 msec at any time prior to cycle 7) | |
| PALLAS | Screening /// C1D14 visit for patients randomized to Arm A /// any time, if clinically indicated | Screening Phase /// Any time, if clinically indicated |
| PALOMA-2 | C1D1 /// C1D14 /// C2D14 /// C4D1 /// C7D1 /// C10D1. ECGs beyond Cycle 10 were performed as indicated clinically. | |
| PALOMA-3 | 28 days prior to randomization unless specified otherwise /// End of Treatment or Withdrawal | |
| PALOMA-4 | 28 days prior to randomization unless specified otherwise /// C1D1 /// C1D14 /// C2D1 /// C2D14 //// Cycles ≥3 Day 1 /// End of Treatment or Withdrawal | |

C=Cycle(s);D=Day(s); W=Week(s).

Supplementary Figure 11**.** Critical appraisal of individual studies according to the Cochrane Collaboration’s tool for assessing risk of bias in randomized trials.

**
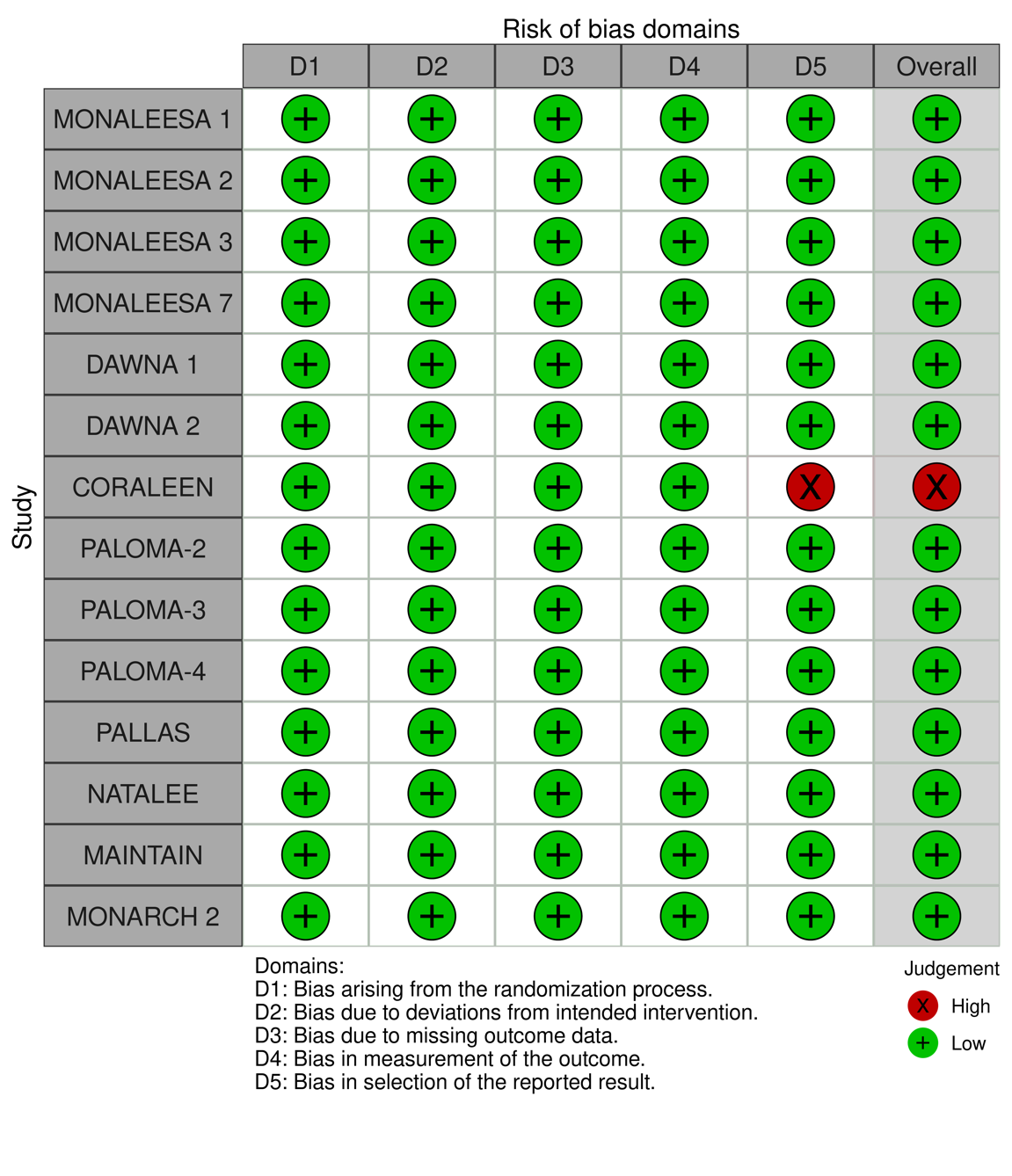
**

Supplementary Figure 12**.** Summary of risk of bias.

**
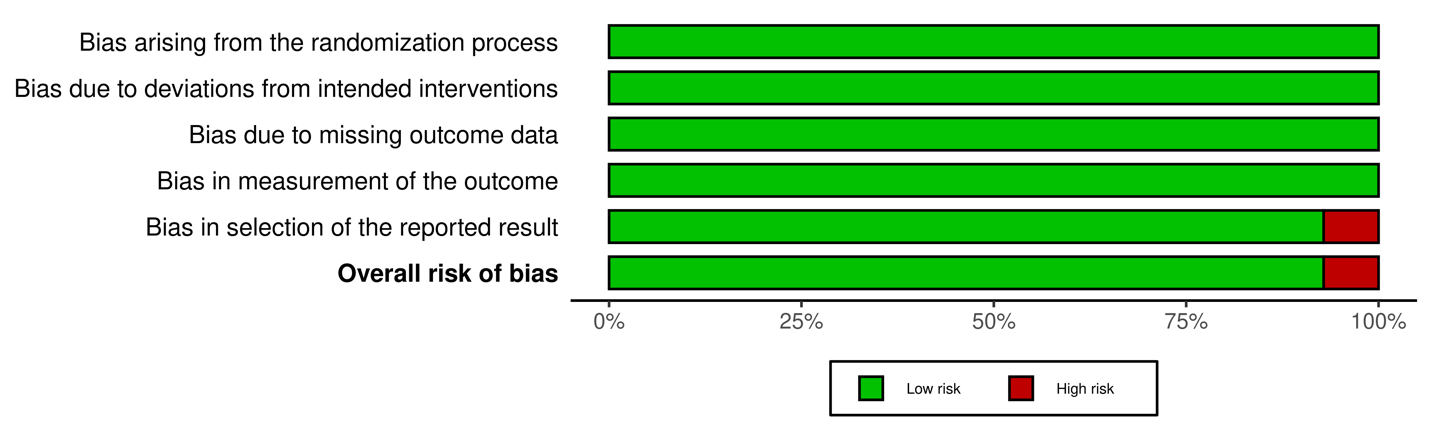
**

Supplementary Figure 13**.** Publication bias assessment by funnel plot for ALL GRADE QTc elevation and Egger´s regression test: t = 0.55, df = 12, p-value = 0.5933.


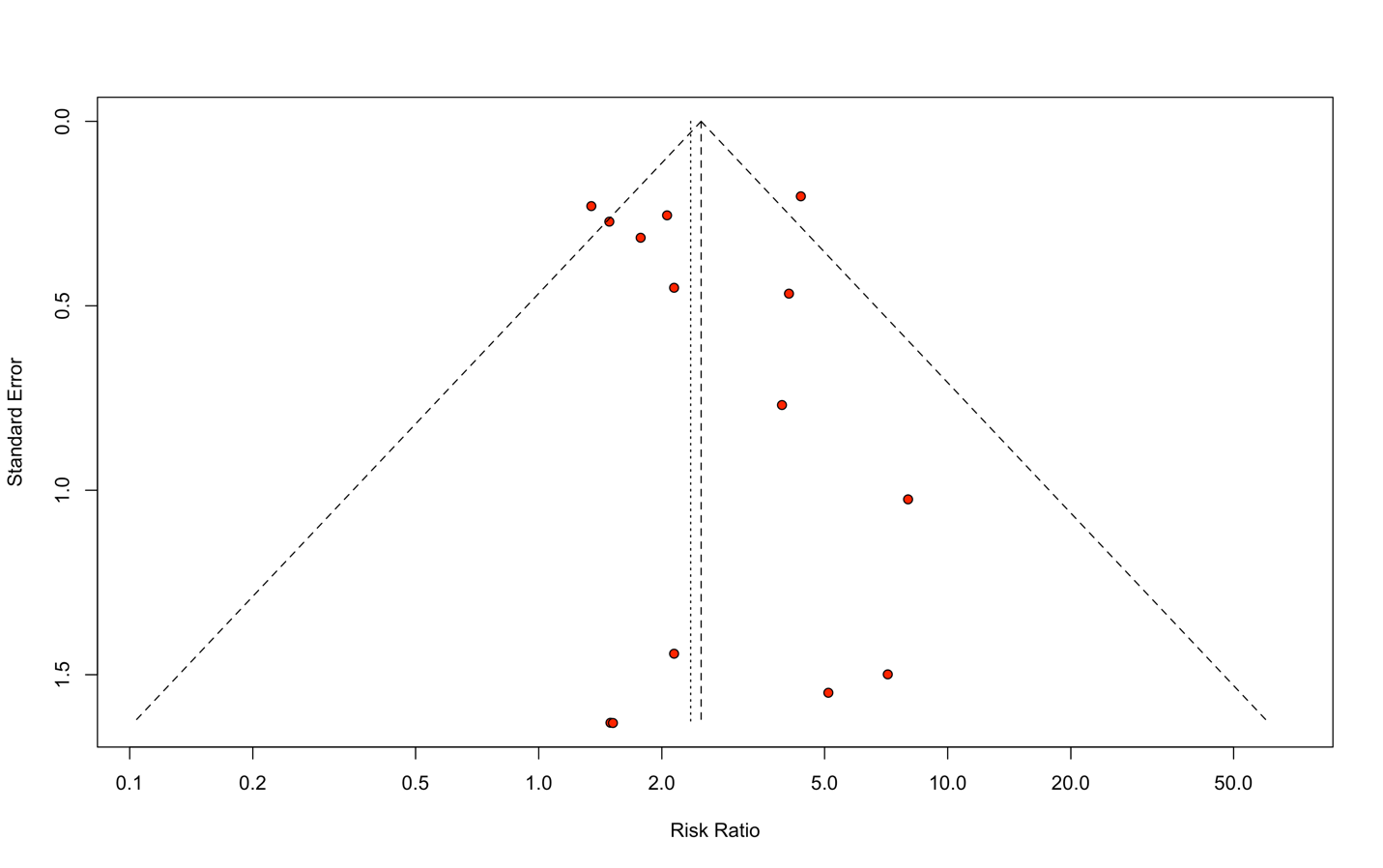

Supplement: pkae078_Supplementary_Data [file pkae078_supplementary_data.docx]
